# Supplementary material for: Effect of Water Supplementation on Oxidant/Antioxidant Activities and Total Phenol Content in Growing Olives of the Morisca and Manzanilla Varieties
Source: Antioxidants (Basel). 2022 Apr 7;11(4):729. doi: 10.3390/antiox11040729 (PMC9024844; doi:10.3390/antiox11040729)
Supplement: Supplementary file 1 [file antioxidants-11-00729-s001.zip › antioxidants-1621287-supplementary.pdf]

Table S1. Mean (maximum and minimum) temperatures, evapotranspiration (ETo), and monthly rainfall (NI) and monthly rainfall plus supplementary irrigation (FI) during 2011, 2012, and 2013 at the study location.

	Max temp (ºC)			Min temp (ºC)			ETo (mm)			Rainfall (NI)/ **Irrigation*** (**FI**) (mm)		
	2011	2012	2013	2011	2012	2013	2011	2012	2013	2011	2012	2013
January	12.26	14.23	13.06	4.17	1.26	4.57	31.12	35.64	32.46	33.60	15.64	40.60
February	15.85	14.52	13.10	3.74	-1.04	2.51	53.22	63.51	46.31	27.80	1.19	77.00
March	16.63	20.27	15.07	6.30	5.44	6.97	75.99	108.60	66.19	57.34	2.18	104.20
April	23.61	17.13	20.19	10.94	6.86	7.55	121.95	99.10	114.47	37.82/**62.46**	53.86/**86.62**	23.60/**37.26**
May	26.88	27.20	23.49	13.70	12.87	9.39	153.94	171.95	154.34	23.96/**62.86**	20.99/**65.60**	16.40/**73.26**
June	30.83	31.27	29.09	14.70	15.53	14.23	197.24	205.86	182.57	6.73/**56.31**	0.00/**55.42**	10.80/**50.88**
July	32.99	33.85	34.72	16.66	16.41	18.20	223.70	225.34	216.96	0.00/**62.12**	0.00/**61.57**	0.80/**57.66**
August	32.90	34.37	34.83	17.47	17.30	18.21	185.62	197.33	195.12	21.38/**82.55**	0.00/**61.34**	1.60/**42.44**
September	30.29	29.00	30.40	15.07	15.63	16.47	132.39	127.17	138.23	11.29/**41.48**	31.50/**71.75**	12.00/**46.62**
October	26.56	22.23	23.57	12.08	11.16	11.70	99.82	77.51	79.52	59.80/**82.20**	84.60/**101.17**	68.20/**84.83**
November	16.95	15.68	15.40	7.73	7.57	4.03	45.21	36.95	39.83	64.35	148.20	4.60
December	13.28	13.71	14.51	3.13	4.59	2.75	28.60	27.17	36.34	10.49	32.60	44.60
Mean temp	23.25	22.78	22.28	10.47	9.46	9.71						
Total rainfall (NI)										354.56	390.76	404.40
Total rainfall+irrigation (**FI**)										**643.56**	**703.28**	**663.75**
Total ETo							1348.80	1376.13	1302.34			

** The total of rainfall plus irrigation if applied.*

Table S2. Fruit yield, oil concentration and oil yield of olive trees for each cultivar, treatment and year.

Year	cv	I	Fruit yield (kg ha^{-1})	Oil yield (kg ha^{-1})	Oil concentration (% DW)
2011	MOR	NI	4686.3	1874.5	40.0
		FI	6952.4	3100.7	44.6
	MAN	NI	5886.4	2713.6	46.1
		FI	7758.5	3173.2	40.9
2012	MOR	NI	3448.0	1427.5	41.4
		FI	5390.7	2301.8	42.7
	MAN	NI	4991.3	2395.8	48.0
		FI	5365.5	2527.1	47.1

2013	MOR	NI	1905.0	963.9	50.6
		FI	3602.2	1700.2	47.2
	MAN	NI	4953.0	2273.4	45.9
		FI	7002.8	3088.2	44.1

MON: cv. Morisca; MAN: cv. Manzanilla de Sevilla; I: water regime; NI: rainfed; FI: irrigation.

Table S3. Biochemical parameters corresponding to olive fruits harvested in 2011, 2012, and 2013 (See M&M). Total soluble amino acids and total proteins expressed as mg g^{-1} FW; NADH oxidation, $O_2^{\cdot-}$ production and POX activity expressed as nmoles min^{-1} mg^{-1} protein; SOD and PPO activities was expressed as U mg^{-1} protein; total phenols, total flavonoids, PPGS and total FRAP expressed as μg g^{-1} FW.

Year	cv	I	R	DW/FW	Total soluble amino acids	Total protein	NADH oxidation	$O_2^{\cdot-}$ production	SOD	POX	Total Phenols	Total flavonoids	Total PPGs	PPO	FRAP
2011	MOR	NI	S1	0.264±0.022	3321.6±301.5	0.963±0.100	354.9±84.6	309.9±59.9	42.5±9.9	60.2±4.9	1671.2±55.4	4356.5±324.6	7269.5±513.0	1080.1±136.8	21.7±0.8
			S2	0.286±0.030	3503.5±331.0	1.271±0.040	596.4±160.5	142.7±59.7	50.8±9.0	161.8±35.5	1719.1±261.0	4284.2±497.0	6942.7±191.9	298.8±33.7	26.7±0.6
			S3	0.347±0.050	6055.7±65.4	3.380±0.400	422.0±137.0	207.6±61.0	43.9±7.3	60.8±13.0	2213.6±39.3	6874.6±181.1	9305.7±1787.9	379.9±70.1	27.9±0.4
		FI	S1	0.300±0.020	2761.2±430.7	1.009±0.090	248.9±25.2	148.0±33.5	44.9±9.5	32.6±4.7	1746.0±51.6	5171.0±146.6	7761.5±550.8	1026.5±193.9	21.5±0.9
			S2	0.265±0.030	2319.7±409.0	1.150±0.200	452.5±22.4	158.9±25.2	49.3±7.9	101.8±9.6	1726.1±12.3	4642.4±529.3	6215.9±498.7	718.4±75.2	26.5±0.6
			S3	0.318±0.020	5423.7±204.0	3.750±0.500	506.7±51.0	280.1±81.8	58.4±17.9	98.0±21.0	2221.6±14.1	7023.2±65.8	9894.0±860.7	336.3±29.8	28.0±0.3
	MAN	NI	S1	0.351±0.033	2087.0±324.0	0.652±0.130	904.8±130.8	393.2±50.3	140.7±18.5	187.4±17.1	1680.7±73.2	4448.4±331.5	7162.5±423.5	666.7±105.5	22.0±1.0
			S2	0.289±0.010	1015.8±149.0	1.067±0.160	688.7±43.5	355.7±23.7	58.5±10.4	124.7±9.6	2002.5±75.6	5230.3±812.5	7883.4±724.2	349.7±34.1	26.6±0.6
			S3	0.362±0.030	3962.7±73.8	3.360±0.300	252.7±19.0	61.2±17.0	66.3±14.5	51.6±5.2	2217.9±33.7	6997.7±174.5	10285.0±944.7	260.2±66.7	27.9±0.3
		FI	S1	0.324±0.030	2513.2±324.7	0.565±0.060	875.9±153.1	392.8±52.5	135.8±14.3	162.5±12.2	1791.6±44.6	5347.1±115.4	8186.3±322.1	650.8±49.1	22.0±0.8
			S2	0.289±0.020	1115.5±185.3	1.191±0.170	745.3±135.5	202.7±17.5	49.4±11.3	99.9±24.2	1958.2±168.3	5152.3±817.0	6291.6±816.9	462.7±76.9	26.8±0.7
			S3	0.364±0.061	4332.5±187.0	3.070±0.500	541.6±77.9	225.8±87.0	64.9±15.8	79.3±10.0	2226.2±23.7	6966.5±141.7	10275.2±671.9	506.4±72.3	27.8±0.1
2012	MOR	NI	S1	0.380±0.006	2441.6±272.0	1.860±0.170	444.1±46.9	258.6±66.8	67.5±6.9	1096.9±20.8	3984.4±574.6	10430.4±1048.0	16208.4±1217.2	479.5±37.2	100±37.9
			S2	0.318±0.020	2453.8±271.0	2.189±0.300	2079.7±127.0	1053.7±248.0	120.2±10.0	261.0±19.1	2987.5±422.0	6904.1±657.6	11998.3±1274.0	424.6±110.0	98.2±6.9
			S3	0.337±0.020	5239.6±907.9	2.545±0.100	902.6±243.0	433.3±70.0	104.2±10.7	161.9±28.8	4240.2±758.9	9237.1±1549.2	13571.0±1867.7	368.0±45.9	124.4±7.4
		FI	S1	0.295±0.010	1592.0±277.2	1.557±0.180	478.4±64.7	497.7±48.4	60.6±0.5	153.4±18.6	3384.1±456.9	7839.6±976.4	14300.1±1051.8	351.8±57.6	97.0±5.7
			S2	0.259±0.030	4249.3±514.0	2.292±0.100	1144.0±388.0	638.9±169.0	90.5±17.0	255.1±30.4	2049.3±171.0	4875.0±479.0	7095.4±438.4	289.7±11.2	98.7±11.3
			S3	0.300±0.008	5523.0±855.2	2.505±0.100	754.6±239.0	531.3±131.2	92.8±12.4	100.7±16.6	3759.8±1153.1	8462.3±1600.0	10050.2±1765.9	304.8±32.5	110.8±18.3
	MAN	NI	S1	0.324±0.015	1428.7±399.6	0.898±0.160	787.5±73.4	492.6±70.2	162.1±14.1	205.7±31.4	3263.9±364.8	7960.9±996.7	13187.8±1421.3	352.9±63.3	127.5±11.5
			S2	0.341±0.008	1598.5±53.6	0.982±0.070	1034.7±205.0	427.2±101.0	226.0±5.7	143.3±28.0	1999.6±307.0	5568.6±338.9	10955.1±1362.5	296.6±57.5	118.6±9.6
			S3	0.292±0.014	2538.7±728.3	1.960±0.170	916.7±206.8	381.8±51.3	141.6±15.7	99.9±19.2	4077.1±327.6	8799.3±860.6	13867.0±1381.2	350.9±39.0	125.4±12.1
		FI	S1	0.303±0.010	2484.3±239.5	0.759±0.210	390.7±67.5	319.6±52.3	171.7±29.6	167.3±38.7	2920.6±683.5	5776.8±517.5	10998.5±1122.3	341.3±31.9	103.0±10.6
			S2	0.262±0.020	3185.0±298.0	1.292±0.100	955.1±60.0	327.3±79.0	187.1±37.0	160.7±15.0	2257.7±193.0	5178.1±320.9	10468.1±971.9	276.4±32.5	97.7±5.7
			S3	0.278±0.015	2541.7±234.2	2.160±0.380	856.8±102.8	341.1±49.6	155.9±20.9	73.6±23.8	4323.5±362.1	7895.0±673.1	10892.1±1663.6	475.5±49.1	135.9±16.3
2013	MOR	NI	S1	0.290±0.022	1843.6±258.1	1.449±0.210	386.1±121.8	251.9±29.7	41.9±7.9	77.1±9.1	3825.9±249.8	8901.0±438.4	15009.9±860.3	455.3±49.9	109.0±21.1
			S2	0.312±0.020	2296.7±307.5	2.058±0.280	1146.8±183.8	333.6±72.1	128.3±14.6	146.9±36.0	3369.4±546.4	8444.3±755.2	12134.0±602.1	280.2±23.6	92.6±15.8
			S3	0.316±0.019	2102.0±150.1	1.947±0.210	186.9±29.3	104.1±34.8	129.6±14.9	21.1±1.9	3398.9±142.2	7067.3±760.0	11705.5±1567.7	354.6±56.8	106.1±14.0
		FI	S1	0.270±0.017	1604.6±180.6	1.366±0.150	324.5±25.7	252.1±50.2	50.7±7.8	56.2±8.1	3673.2±418.6	5539.1±231.8	13527.9±1271.7	424.1±35.2	98.9±11.1
			S2	0.296±0.020	2125.5±330.9	2.198±0.210	677.9±107.4	273.3±25.1	105.2±10.0	165.5±5.7	2854.5±412.0	5629.5±983.5	13134.6±424.2	250.6±56.3	69.1±8.8
			S3	0.274±0.017	3153.5±298.9	2.013±0.260	1206.0±191.0	432.0±66.1	109.6±18.7	158.4±15.8	3230.6±97.7	6182.8±301.8	9332.2±956.5	306.8±45.3	85.1±5.9

	MAN	NI	S1	0.309±0.028	1228.7±146.3	0.628±0.090	308.1±37.5	294.3±58.1	114.0±17.5	52.4±11.2	2937.2±389.1	5477.6±251.2	10760.8±961.7	249.0±54.3	91.6±11.3
			S2	0.299±0.030	1778.5±281.6	1.487±0.080	1272.4±225.0	358.8±74.9	208.1±15.0	180.6±32.2	2817.6±140.0	6252.6±781.0	12566.9±1014.4	215.2±18.7	115.9±17.5
			S3	0.317±0.029	1977.7±277.3	1.848±0.250	624.5±50.2	240.3±45.9	213.0±33.9	71.6±10.9	3598.9±108.7	6849.0±291.3	11899.8±172.7	313.8±61.9	120.5±20.3
		FI	S1	0.252±0.006	1139.1±216.4	0.576±0.140	345.5±74.0	238.9±32.0	110.7±18.0	46.2±12.3	3347.7±638.9	6447.8±569.1	12304.3±1111.2	258.0±38.6	108.7±8.3
			S2	0.228±0.020	975.9±186.6	1.266±0.240	749.1±182.0	272.4±50.4	142.3±12.8	177.9±21.3	2687.5±98.0	4739.0±359.0	9189.6±946.9	182.7±48.8	66.7±14.2
			S3	0.285±0.015	216.4±273.5	2.077±0.160	717.6±115.0	219.6±65.0	195.9±11.3	65.7±13.6	3296.3±67.9	6218.9±265.7	8791.5±565.6	323.9±56.8	95.7±17.3

MON: cv. Morisca; MAN: cv. Manzanilla de Sevilla; I: water regime; NI: rainfed; FI: irrigation; R: ripening stage.
